# Pseudohypertension-Like Presentation: An Exceptionally Rare Presentation in an Athletic Female Patient with Morphea

**DOI:** 10.1155/2016/7027352

**Published:** 2016-12-29

**Authors:** Ahmed Al-Imam

**Affiliations:** ^1^Novel Psychoactive Substances Research Unit, University of Hertfordshire Doctoral College, Hertfordshire University, Hatfield, UK; ^2^Faculty of Medicine, University of Baghdad, Baghdad, Iraq

## Abstract

*Introduction*. Pseudohypertension is a condition which mainly occurs due to thickening-calcification of tunica intima of the arterial wall, leading to a faulty measurement of the intra-arterial blood pressure. To the best of our knowledge, this is the first case report in literature, of a pseudohypertension-like presentation in association with Morphea en plaque.* Case Presentation*. This is a rare presentation of a young athletic female and a professional tennis player, with pseudohypertension-like presentation. The patient had a traumatic injury to the right elbow joint; the injury occurred during a professional tennis match. The injury was managed by immobilization, physiotherapy, and Low-Level Laser Therapy. Soon after that, the patient had a circumscribed sclerotic ivory plaque affecting the skin of the right cubital fossa. The histopathology analysis, together with the serological-hematological tests and the clinical picture, along with positive Osler's signs, leads to the conclusive diagnosis of Morphea en plaque. The peculiar anatomic localization of the plaque anterior to the brachial artery leads to faulty blood pressure measurement as recorded by mercurial sphygmomanometer.* Conclusion*. This unique presentation of Morphea en plaque carries an important message in relation to the basic medical practice and in relation to the accurate measurement of the vital signs.

## 1. Introduction

Pseudohypertension, also known as the noncompressibility artery syndrome, occurs when there is a faulty-recorded elevation of blood pressure in a normal individual or an exaggerated elevation of blood pressure in an already hypertensive individual, as measured via sphygmomanometer. This vascular phenomenon occurs mostly in advanced age due to calcification-thickening of the arterial vascular wall, the tunica intima layer to be specific, which makes the artery difficult to be compressed and/or occluded by the sphygmomanometer. This condition can be clinically tested using Osler's maneuver, and accordingly a patient is to be diagnosed with pseudohypertension when Osler's sign is positive [1, 2]. In this case presentation, a faulty high blood pressure, a pseudohypertension-like phenomenon in a young and athletic female, is reported as a result of a circumscribed plaque of Morphea (Morphea en plaque), developing in the cutaneous tissue overlying the brachial artery in the region of the right cubital fossa. This pathology occurred following a traumatic injury to the right elbow joint, with consequent physiotherapy and management with Low-Level Laser Therapy (LLLT).

This phenomenon results in an elevated cuff pressure compared with intra-arterial measurements and is found primarily in advanced age. In a retrospective study (Kleman et al., 2013), conducted on patient attending a hypertension clinic, it was found that 7% of the patients had pseudohypertension [3]. In a parallel cohort study (Collins et al., 2013), the prevalence of systolic pseudohypertension in patients undergoing Cardiac Catheterization was detected in 10.8% of patients, and the majority were of advanced age [4]. In relation to the clinical examination of pseudohypertension, Osler's maneuver is to be performed for assessing the palpability of pulsation of the radial and/or brachial artery distal to a point of the suspected arterial pathology. Patients who are Osler's sign positive have falsely elevated blood-pressure readings, in the range of 10 to 54 mm Hg between cuff pressure and intra-arterial pressure [5, 6].

**Figure 1:**
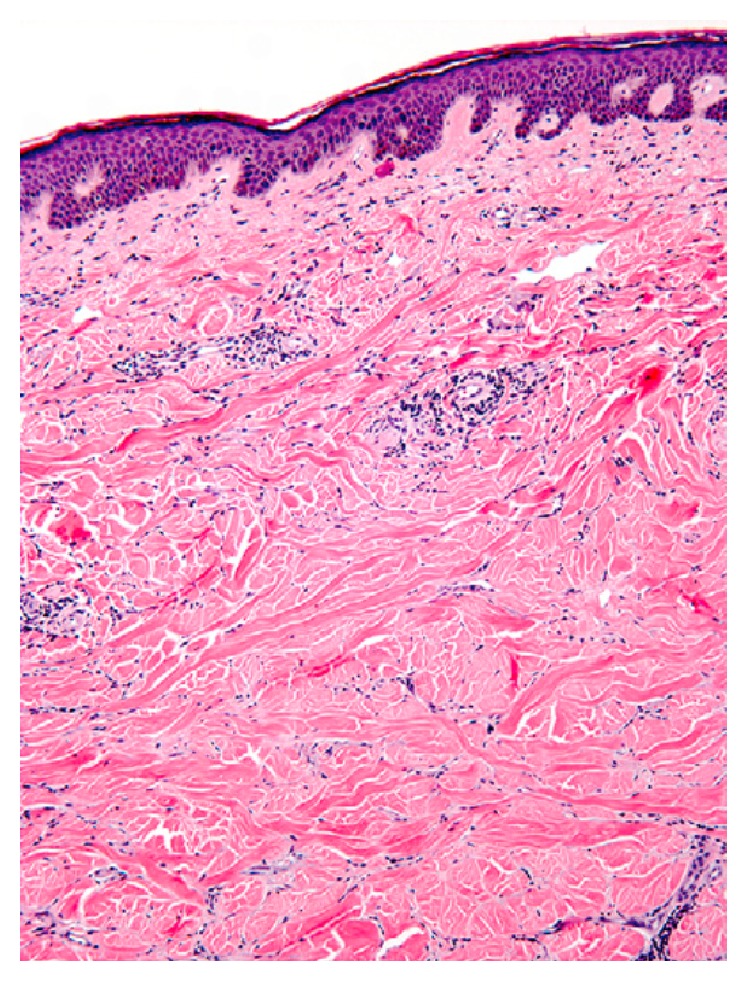
Histology of an early stage of Morphea. Evident swollen collagen bundles, with perivascular lymphocytic infiltrate and plump endothelial cells. Hematoxylin-eosin stain; 10x magnification power.

Morphea is localised form scleroderma, with an estimated incidence of 0.4–2.7 per 100,000 people affecting more females than males [7, 8]. Morphea is almost uniformly limited to those tissues derived from the mesoderm, mainly reticular layer of the dermis. The underlying pathogenesis can be partially attributed to an imbalance of collagen production and destruction, leading to an excess of deposited type-1 and type-3 collagen fibres, as seen in Figure 1 [9].

## 2. Case Presentation

The patient is an eighteen-year-old professional tennis player, is completely healthy, and lacks any family history of hypertension, hyperlipidemia, or other cardiovascular disorders/risks. She has been recently and accidently discovered with hypertensive episodes and was diagnosed by a cardiologist. The hypertensive episodes started approximately five months ago; they were persistent in nature reaching up to 155/100 mmHg as measured by a traditional mercurial sphygmomanometer. However, the patient was symptomless and did not suffer from headaches or other clinical features of hypertension. Besides, early-onset essential hypertension and corresponding comorbidity were investigated using ECG, stress ECG, echocardiography, cardiac risk biochemistry, and lipid profile. However, all were found to be normal.

It is worth mentioning that the patient had a traumatic injury to the right elbow joint, approximately eight months prior to the presentation with the assumed hypertension, the traumatic injury occurred during a tennis match, and the patient was diagnosed with multiple ligaments injury around the elbow and proximal radioulnar joint, specifically the lateral collateral ligament and the annular ligament. The diagnosis of the elbow joint injury was confirmed by clinical examination and radiography using MRI imaging, the patient was advised to limit her daily activities, and the elbow was immobilised by applying plaster of Paris cast. This was later followed by physiotherapy and Low-Level Laser Therapy (LLLT) for the affected (right) elbow. Two months later, the patient presented with an indurated ivory-colored plaque and inflammatory lilac-colored borders; the plaque was localised to the skin of the anterior aspect of the elbow joint and was progressing slowly over that area.

Accordingly, the patient was referred to a specialist dermatologist, who suspected the diagnosis of a localised form of sclerosis and measured the plaque to be of a maximal transverse diameter of 6.52 cm and a vertical diameter of 5.37 cm; these measurements were documented using a digital Vernier Caliper. Further, he took a deep punch biopsy from the ivory-colored central part and another punch from the peripheral (active inflammatory) edge of the plaque. The pathologist report confirmed the presence of homogeneous bundles of collagen fibres, specifically type-1 and type-3, confined to the reticular layer of the dermis (Figure 1); the upper part of the subcutis was completely normal [9]. Other investigations, serological and blood tests, were concordant with the clinicopathological picture, and a diagnosis of Morphea en plaque was established. Positive serologic markers included homogeneous ANA, anti-single-stranded antibody, anti-topoisomerase II-*α* antibody, rheumatoid factor, and anti-Fc-*γ* receptor antibody.

The morphoeic plaque was localised exactly over the area of the brachial artery, and, in relation to the anatomical site of the cubital fossa, the plaque was more extensive in the lateral (transverse) direction than vertically. From a biomechanical perspective, this idiosyncratic anatomical position of the plaque induced a sclerotic shield-like covering over the brachial artery rendering it incompressible at normal pressure exerted by the sphygmomanometer as compared to the contralateral brachial artery. Further, this was consolidated by positive Osler's sign, when palpating the more distal radial artery.

Accordingly, the patient was instructed to stop her antihypertensive medications, and her dermatologist started managing the solitary and localised morphoeic plaque, using superpotent topical steroids, Dermovate (clobetasol propionate) ointment applied twice daily to the lesion. The patient started to show an excellent response, the inflammatory edge started to regress, and the plaque was no longer expanding in size, and the patient was assured that the lesion would regress completely within the next years, with a low possibility of progression to a more generalised form of scleroderma.

This presentation is exceptional; in a patient of Morphea, the initial wrong diagnosis of hypertension was based on mere metric data of a classic sphygmomanometer. Unfortunately, the cardiologist relied on measuring the blood pressure only from the right brachial artery. Furthermore, pulse rate measurement and examination of characteristics of the radial pulse have been checked by the cardiologist including radial pulse, volume, and the status of the blood vessel wall. However, no abnormalities were reported. The effective collaboration and proper attention to the patient dermatologic condition and within the context of interdisciplinary thinking of clinical medicine lead to the proper diagnosis and management of this young and athletic female patient.

The level of evidence of this manuscript is level 5, according to the Oxford Centre for Evidence-Based Medicine—Levels of Evidence [10]. The literature review was done across PubMed, the Cochrane Library, Scopus, and Google Scholar. The search strategy was based on a keyword list applied to these medical databases; the keywords used were as follows: “Pseudohypertension”; “Arterial Pressure”; “Osler's sign”; “Localised Scleroderma”; “Morphea”; “Collagen”; and “Low-Level Light Therapy”. A combination of these keywords was also applied using* Boolean Operators* [11]. Surprisingly, only a single published manuscript appeared, when the keywords “Pseudohypertension AND Morphea” were used, an indicator of the extreme paucity of the published scientific literature [12].

## 3. Discussion

This is an exceptionally rare case presentation of a young and an athletic female; she has been wrongly diagnosed with hypertension. Later, a proper diagnosis of Morphea and pseudohypertension-like presentation was concluded. Pseudohypertension usually occurs in advanced age, unlike this presented case.

The morphoeic lesion was anatomically localised in relation to the right cubital fossa, the skin that is immediately in front of the brachial artery. The patient correlated the evolution of her skin lesion with a traumatic injury of her right elbow, for which she was treated with physiotherapy and Low-Level Laser Therapy.

The author insight into the evolution of the morphoeic might be correlated with the use of LLLT. Low-Level Laser Therapy was used for this patient as a therapeutic modality to rehabilitate and alleviate pain, to restore the traumatised right elbow joint back to functionality. In the literature, it is documented that LLLT can significantly reduce pain and improves health status in chronic joint disorders, but the disparity in patient samples, treatment procedures, and trial design led to unconfirmed and heterogeneous level of evidence [13]. Similarly, there is a significant paucity of verified evidence in the scientific literature, to conclude whether LLLT might induce or be associated with triggering Morphea in the skin overlying the treated areas.

The keen interdisciplinary approach of the cardiologist, dermatologist, and the dermatopathologist leads to the proper diagnosis and the subsequent successful management of this patient. The mistake committed by the cardiologist was to rely on the measurement of the arterial blood pressure of right brachial artery (unilaterally) while omitting and failing to compare the pressure bilaterally and with the distal radial pulse, representing a basic and important message to all physicians, medical practitioners, and the paramedical staff.
